# Insights into the Inhibitory Mechanisms of the Covalent Drugs for DNMT3A

**DOI:** 10.3390/ijms241612652

**Published:** 2023-08-10

**Authors:** Wei Yang, Jingyuan Zhuang, Chen Li, Chen Bai, Guijuan Cheng

**Affiliations:** 1 Warshel Institute for Computational Biology, School of Medicine, The Chinese University of Hong Kong, Shenzhen 518172, China; 2 National Clinical Research Center for Infectious Diseases, Shenzhen Third People’s Hospital, Shenzhen 518112, China; 3 Biomedicine Discovery Institute, Department of Biochemistry and Molecular Biology, Monash University, Melbourne, VIC 3800, Australia; chen.li@monash.edu; 4 School of Life and Health Sciences, The Chinese University of Hong Kong, Shenzhen 518172, China; 5 School of Medicine, The Chinese University of Hong Kong, Shenzhen 518172, China; 6 Shenzhen Futian Biomedical Innovation R&D Center, The Chinese University of Hong Kong, Shenzhen 518017, China

**Keywords:** DNMT3A, QM/MM, covalent drugs, reaction mechanism

## Abstract

The perturbations of DNA methyltransferase 3 alpha (DNMT3A) may cause uncontrolled gene expression, resulting in cancers and tumors. The DNMT inhibitors Azacytidine (AZA) and Zebularine (ZEB) inhibit the DNMT family with no specificities, and consequently would bring side effects during the treatment. Therefore, it is vital to understand the inhibitory mechanisms in DNMT3A to inform the new inhibitor design for DNMTs. Herein, we carried out molecular dynamics (MD) and quantum mechanics/molecular mechanics (QM/MM) simulations to investigate the inhibitory mechanisms of the AZA and ZEB. The results were compared to the methyl transfer of cytosine. We showed how the AZA might stop the methyl transfer process, whereas the ZEB might be stuck in a methyl-transferred intermediate (IM3). The IM3 state then fails the elimination due to the unique protein dynamics that result in missing the catalytic water chain. Our results brought atomic-level insights into the mechanisms of the two drugs in DNMT3A, which could benefit the new generation of drug design for the DNMTs.

## 1. Introduction

DNMTs are a family of essential epigenetic modifiers that play a fundamental role in numerous cell and development processes [1,2]. Their basic mechanism is implemented via the transfer of a methyl group from the S-adenosyl-L-methionine (SAM) molecule to the C5 position of the cytosine (dC) in the CpG islands [3]. Once the CpG islands are labeled with a methyl group, the gene is silenced. Therefore, the expression levels of DNMTs are directly related to the silencing and expression of the epigenetic transgenes, and therefore are reported to elevate cancers in many organs, e.g., colon [4], prostate [5], breast [6], liver [7,8], and blood (i.e., leukemia) [9,10]. Four mammalian DNA methyltransferases have been identified to date, including DNMT1, DNMT3A/B, and DNMT2 [9]. It has been reported that DNMT3A/B acts as de novo methyltransferases and sets the whole epigenetic pattern of the DNA [11]. DNA replication without the methyl label would create a new complement chain. The DNMT1 then adds the methyl group to the DNA daughter strand, thus acting as a maintenance DNMT. Comparing the clear biochemical roles of DNMT1, the role of DNMT2 is still under debate [9].

**Figure 1:**
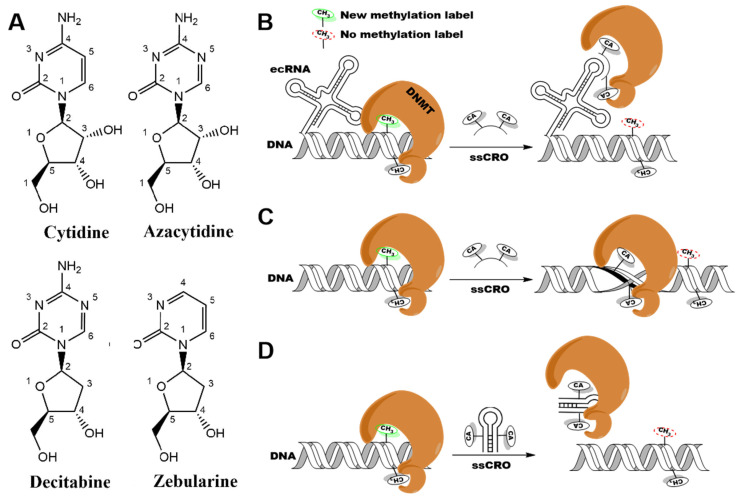
The nucleoside DNMT inhibitors were used to assemble more complex chimeric RNA oligo-nucleotides (ssCRO or dsCRO) for the different inhibitory strategies towards DNMTs. (**A**) Chemical structures of Cytidine (dC), Azacytidine (AZA), Decitabine (DEC), and Zebularine (ZEB), respectively. (**B**) The ssCRO forms a double-stranded complex with a natural RNA for DNMT inhibition. (**C**) The ssCRO forms a duplex structure with genomic DNA for inhibiting DNMT. (**D**) The DNMT sequestration mechanism of dsCRO. The CA refers to the cytosine analogs such as AZA, DEC, ZEB, etc. The C refers to Cytidine.

The overexpression and mutations of the DNMTs are closely related to oncogenic activation. They have been validated as drug targets for series cancer and tumors [12]. To date, several strategies to inhibit DNMT have been developed and reviewed [13]. These strategies can be concluded as nucleoside and non-nucleoside analog compounds, acting as competitors of the target cytidine, and preventing the methyl transfer process in various ways [13]. To date, the number of nucleotide analogs available on the market has increased to more than 30 as an effective therapeutic strategy against multiple types of infections (e.g., viral, bacterial, and fungal infections), parasites, and cancers [14,15]. Two successful examples of cytidine analogs are 5-Azacytidine (or azacitidine, AZA) and 5-aza-2′-deoxycytidine (or decitabine, DEC) (Figure 1A). Approved by the USA Food and Drug Administration (FDA) and the European Medicines Agency (EMA), both have been used for medical treatment in acute myeloid leukemia (AML), chronic myelomonocytic leukemia (CMML), and myelodysplastic syndromes (MDS) [16,17]. Another more stable cytidine analog is Zebularine (ZEB) (Figure 1A), which is usually used in the co-crystallization of DNMTs X-ray structures [18]. The ZEB has been found to function at high doses in cell experiments [19], but failed in mice experiments that might be related to different genders [20]. These cytidine analogs were usually used to incorporate into the single or double strand(s) chimeric RNA oligo-nucleotides (ssCRO or dsCRO) (US20140171492, WO2014011573, and WO2012142480), which were capable of selecting the target sequences or specifically hybridizing the target genomes and then silencing the gene by chelating the DNMTs (Figure 1B–D). The ssCROs carried complementary base sequences to a small amount (usually with ~80% of 15 to 30 bp) of an extra-coding RNA (ecRNA, as shown in Figure 1B). They can silence the DNMT1 by forming a double-stranded complex with the natural ecRNA (Figure 1B). Another circumstance is that ssCRO formed a duplex structure by hybridizing the genomic DNA sequences (Figure 1C). The dsCROs generally sequestrated the DNMTs by forming a DNMT-dsCRO silence complex, as shown in Figure 1D. The cytidine analogs of these CROs (e.g., AZA, DEC, 5-fluoro-cytidine, fluoro-cyclopentenyl-cytosine, ZEB, and deoxy-ZEB, etc.) were supposed to covalently or non-covalently bind the DNMTs and thus, in turn, lead the enzymes to degradation and reduced DNA methylation of the target gene [21,22,23]. Compared to AZA (Azacytidine) and DEC (Decitabine), ZEB is more stable in aqueous solutions [15]. The ZEB has higher specificity in some types of cancer cells with lower general toxicity compared to the AZA and DEC [19]. Additionally, the ZEB outperformed the AZA in terms of decreasing the level of methyl transfer in in vitro blastocyst experiments, thereby better controlling the proliferation of cancer cells [19]. In sum, these inhibitors are highly potent and active, but have poor chemical and metabolic stability and low specificity to different DNMTs when using Cytidine analog alone, thus inducing several side effects [15,20,21,24,25,26]. The less toxic non-nucleoside compounds with different chemical scaffolds have been developed using in silico and experimental screening assays [27,28,29,30,31]. However, it has been reported that only numeral non-nucleoside inhibitors of DNMTs have been developed [13,31,32,33,34]. They either suffer from weak binding or poor selectivity, making it challenging to envisage the structural activity relationship (SAR) [13].

The mechanism-based drug discovery for non-nucleoside small inhibitions has been conducted to improve the potency and selectivity of DNMTs. However, it is still in its infancy due to the lack of studies on complicated catalytical mechanisms of the enzymes with detailed atomic-level understanding [13]. The methyl transfer and inhibition mechanism of the inhibitors to M.H*ha*I DNMT have been studied [35,36]. In the case of DNMT3A, the critical step of methyl transfer was studied previously [37]. Our group has just published the entry methyl transfer mechanism of the DNMT3A in its biological assembly [38]. Recently, utilizing the transition state (TS) structures from the above studies, a new series of inhibitors have been synthesized showing the effective potency of DNMT1 and DNMT3B in the micro-mol range [39]. Given that DNMT3A/B can serve as an oncogene and a tumor suppressor gene in the lung cancer [40], the new inhibitors with selectivity toward DNMT1 and DNMT3A/B should be particularly highlighted in the new drug design of DNMT inhibitors (DNMTi). Hence, an urgent need exists to unveil the inhibitory mechanism of the ZEB and AZA toward DNMT3A and its differences from that of DNMT1. Additionally, the ZEB was believed to share a similar mechanism to the AZA and DEC. However, the ZEB has indeed failed in animal experiments in the preclinical trial due to the significantly different effects on male and female mice [20], but it still showed a stronger ability in decreasing the level of methyl transfer in cell experiments [19]. Therefore, the differences underlying the inhibitory mechanisms between the ZEB and the formers are still undiscovered. In this study, we have presented fully atomic-level hybrid Quantum Mechanics/Molecular Mechanics (QM/MM) and classical Molecular Dynamics (MD) simulations to discover the inhibitory mechanisms of the AZA and ZEB in DNMT3A by the comparison of the dC. Our results provided a theoretical inhibitory explanation of the covalent drugs in DNMT3A, which would hopefully better facilitate the new drug design of DNMTi.

## 2. Result

### 2.1. The Characterization of the Pre-Reaction State

**Figure 2:**
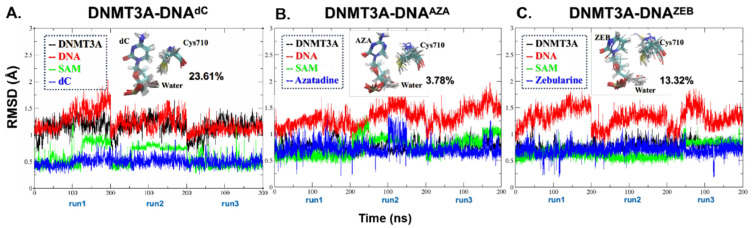
The RMSD and PRS clusters of (**A**) DNMT3A-DNA^dC^, (**B**) DNMT3A-DNA^AZA^ and (**C**) DNMT3A-DNA^ZEB^ MD systems. The RMSD of DNMT3A and DNA were calculated with backbone atoms. The SAM and the flipped-out substrates were calculated on all heavy atoms without hydrogens. The PRS clusters of each system were shown per panel. The calculated percentage of the cluster compared to the total conformational space was listed adjacent to each PRS cluster.

First, we modeled the DNMT3A-DNA systems, in which the flipped-out substrates, dC and AZA, were generated from the ZEB molecule of the crystal structure [41]. The C6^ZEB^-SH^cys710^ (S-C bond) already existed in the crystal structure, owing to the co-crystallization experiment. Thus, the S-C bond was set to broken form to mimic the initial state in which an S-C nucleophilic attack has not occurred in the systems. The thiol group of Cys710 was modeled back to protonated form in each system. We then performed MD simulations on these systems (DNMT3A-DNA^dC^, DNMT3A-DNA^AZA,^ and DNMT3A-DNA^ZEB^) to explore the conformational space for characterizing the pre-reaction states (PRS). Firstly, the system plasticity of DNMT3A, DNA, and the target dC304, AZA, and ZEB molecules were checked with RMSD analysis. The methyl transfer domain of DNMT3A in the DNMT3A-DNA^dC^ was stabilized at 1.47 ± 0.13 Å during the last 100 ns simulations of each replica. On the contrary, the corresponding parts in the inhibitor-bound systems were less fluctuated (around 0.93 ± 0.12 Å in the AZA system and 0.89 ± 0.09 Å in the ZEB system). The heavy atoms in the substrates (dC304, AZA, ZEB), as well as the SAM molecule, experienced similar turbulence (0.63 to 0.76 Å), and were therefore very stable during the MD simulations (Figure 2). More fluctuations (2.12 to 2.55 Å) were observed in the DNA parts (Figure 2). This can be explained by the exposure of the 3′ and 5′ ends of the DNA to the solvent. Above all, these results suggested that the simulated systems were all stable.

**Figure 3:**
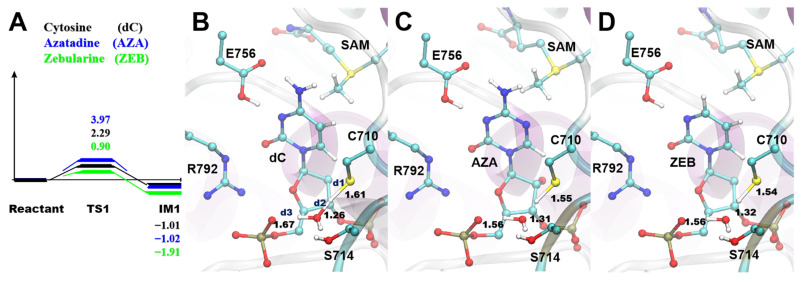
The similar Cys710 deprotonation pathway in DNMT3A-DNA^dC^, DNMT3A-DNA^AZA^, and DNMT3A-DNA^ZEB^ systems. (**A**) The reaction coordinates (RC) of the Cys deprotonation pathway. The total energies of QM/MM were annotated aside by the TS1 and IM1. (**B**–**D**) The zoomed-in structural information of the TS structures in the three systems. The key residues were shown as sticks, and the atoms involved in reactions were displayed as spheres and sticks. The protein and DNA were presented in the cartoon representation.

The PRSs were further narrowed down from the conformations of the above equilibrium production (the last 50ns in each replica of the systems). Former theoretical studies on DNMT1 and M.H*ha*I DNMT indicated that Cys (Cys710) could deliver the hydrogen directly to the oxygen group (OP1 or OP2) of the flipped-out cytosine (dC304) [42,43]. Later, Yang et al. [44] and Aranda et al. [35] declared that water or a nearby Ser could lower activation energy (Ea) in the DNMT1 isomer. Therefore, we supposed the substrates in DNMT3A would also follow the same pathway to initiate the Cys deprotonation process. We clustered the snapshots to make sure a water molecule was located in one bond length (1.2 to 1.8 Å) between the SH atom of C710, OP1 of the AZA/ZEB, and H atom S714 (Figure 3, Supporting Coordinate File (CF)). The clustered snapshots were superimposed in Figure 2 to represent the PRS. We found that the population of the possible PRS took 23.61%, 3.78%, and 13.32% of all the dC, AZA, and ZEB systems, respectively. It indicated that PRS in the dC-containing systems could be achieved more easily than in inhibitor-bounding systems.

### 2.2. Deprotonation of the Cys

It was reported that the series of reactions start from the deprotonation of the conserved Cys residue near the flipped cytosine [3]. The Cys deprotonation was also believed to be the first step in the inhibition mechanism of the covalent inhibitor-containing systems [22]. Therefore, we thoroughly studied the detailed inhibition mechanism, starting from the Cys deprotonation process by QM/MM calculations, based on the representative structure clustered from the former MD systems (Figure 2). Notably, during the nucleophilic attack of DNMT1 and DNMT3A, the protonation of N3 by the conserved Glu (Glu756 in DNMT3A) can activate the aromatic ring of the dC with a lower activation energy barrier [35,36] than the energy barrier from the two reactions occurring one by one. Secondly, our previous study found that the stabilization effect of the thiolate group on H_3_O^+^ can decrease the activation energy as a water molecule is formed during the S-C attack [38]. Given that a concerted reaction for the changing of the three bonds possessed the lowest energy of activation in this step, we only calculated the TS structures of the concerted reaction alone in this work.

The calculated energy of activation (Ea) of the deprotonation step for DNA^ZEB^ was lower (0.90 kcal/mol for DNA^ZEB^) than the other two systems (3.97 kcal/mol for DNA^AZA^ and 2.29 kcal/mol for DNA^dC^; Figure 3 and Supporting CF). The IM1s of the systems were all around −1 kcal/mol. For instance, we noticed that IM1 of DNA^AZA^ and DNA^ZEB^ were even more stable (−1.02 kcal/mol for DNA^AZA^ and −1.11 kcal/mol for DNA^ZEB^) than their PRS structures (−1.01 kcal/mol in DNA^dC^). It indicates that the process could occur spontaneously. The TS1 structures are similar to each other. In the TS1 of the DNMT3A-DNA^dC^ system, the SH of Cys710 (SH^Cys^) is 1.61 Å (d1) to the SG of Cys710 (SG^Cys^). With that distance, it left 1.26 Å (d2) from the SH^Cys^ to the oxygen of the catalytic water (O^WAT^). By contrast, d1 was shortened to 1.55 and 1.54 Å, and d2 is elongated to 1.31 and 1.32 Å in the AZA and ZEB systems, respectively. After TS1, a hydronium ion was generated with H1 of the catalytic water (H1^WAT^) pointing closely (d3 = 1.44, 1.35, and 1.36 Å in the dC, AZA, and ZEB systems) to the oxygen atom (OP1) on the phosphate group of the substrate. Hence, the thiol group was generated by the formation of an H_3_O^+^ in the Cys deprotonation process of each system.

### 2.3. The S-C Attack

**Figure 4:**
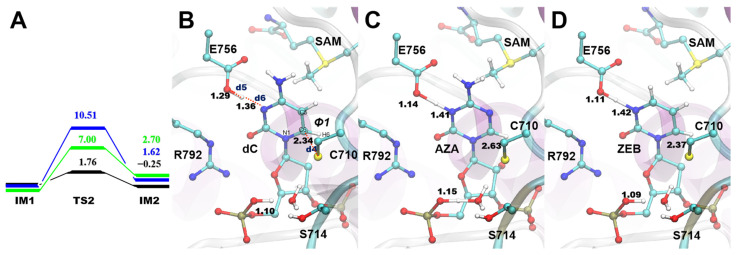
The S-C attack pathway in the three systems. (**A**) The RC of the S-C attack pathway. (**B**–**D**) The detailed structural information of the TS structures in dC, AZA, and ZEB system, respectively.

In the second step, the nucleophilic attack would occur on the C6 atom (C6^dC^, C6^AZA^, C6^ZEB^, respectively) of the substrates by the thiol group of Cys (viz, S-C attack). The activation energy (Ea) of the ZEB system is lower (7.00 kcal/mol, Figure 4A, Supporting CF) than the AZA system (10.51 kcal/mol). The Ea of the dC-containing system is 1.76 kcal/mol, and the IM2 of that is −0.25 kcal/mol. It renders the S-C attack could happen reversely and promptly. Interestingly, such a process also occurs concertedly with the OP1 and N3 protonation in the three systems. Indeed, the protonation of the OP1 atom makes the SG^cys^ more distant from the catalytic water (Figure 4B–D, Supporting CF). Moreover, the negative thiol group approaches C6 of the more positively charged basic ring, which is caused by the protonation of the N3 atom. Indeed, the d5 and d6 indicated proton transfers to N3. Therefore, the SG^cys^ atoms are 2.34, 2.46, and 2.37 Å to the C6 (d4) in the TS2 structures (Figure 4B–D, Supporting CF). Moreover, the H6 atoms rotate down to the ring plane as the *sp^2^* carbon (C6) changes into *sp^3^*. Such rotation can be observed by the *Φ*1 (H6-C6-C5-N1) of TS2 and IM2 structures (Tables S1–S3). Above all, the N3 protonation was coupled with OP1 protonation during the S-C attack of the three substrates.

### 2.4. Methyl Transfer Process

**Figure 5:**
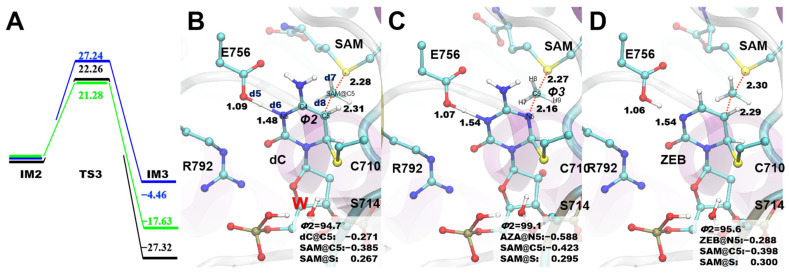
The methyl group transfer pathway in the three systems. (**A**) RC of the methyl transfer pathway. (**B**–**D**) The detailed structural information of the TS structures in the dC (**B**), AZA (**C**), and ZEB (**D**) systems. *Φ*2 and *Φ*3 refer to the dihedral of N3-C4-N5/C5-SAM@C5 and H7-C5-H8-H9, respectively.

In the third step, the methyl group is transferred onto the C5 (dC and ZEB systems) and N5 (AZA system) of the substrates (Figure 5, Supporting CF). As a result, the d7 (distance between the C5 of the residue and C5 of the SAM) and d8 (the distance between S and C5 of the SAM) were nearly in the same length and *Φ*3 (the dihedral of H7, C5, H8, and H9 in the SAM, Figure 5B,C) were all nearly 180° (Tables S1–S3) in the TS3 structures. We found that the Ea in the AZA of this step was very high (27.24 kcal/mol), which poses grave difficulty for the AZA to process the methyl transfer. The barrier for transferring the methyl group in the dC and ZEB systems was much lower (22.26 and 21.28 kcal/mol for the dC and ZEB, respectively). The IM3 in the dC and ZEB systems were also much more stable (−27.32 and −17.63 kcal/mol for the dC and ZEB, respectively) than in the AZA system. It indicated that the ZEB might possess a similar capability to accept the methyl group as the dC, while AZA may not be able to undergo the methyl transfer step. The charge analysis suggested that AZA@N5 (−0.588) carried more negative charges than dC@C5 (−0.271) and ZEB@C5 (−0.288), introducing a stronger electrostatic repulsion with the incoming SAM@C5 atom (−0.423). On the other hand, our DFT studies on the model reaction systems showed that the methyl group prefers to approach the ring of the dC and AZA analogs vertically (*Φ*2 = 94.7°; Figure S1B) and horizontally (158.1 or 123.1°; Figure S1C,D), respectively. Indeed, the methyl groups were also found to approach the ring by nearly 90° in the dC (*Φ*2 = 92.7°) and ZEB-containing (*Φ*2 = 95.6°) QM/MM systems. However, the enzyme constrained the SAM to transfer the methyl group to the base from a vertical direction, resulting in an unfavorable methyl transfer geometry in the AZA system (*Φ*2 = 99.1°; Figure 5C). Thus, both the unfavorable electrostatic interaction and geometry contributed to the higher energy barrier for the methyl transfer process in the AZA system. The methyl transfer process was accompanied by the proton migration from N3 to the conserved Glu756 in all three systems. This proton transfer converted the position of the double bond from C4=C5/N5 to C4=N3. Because the C4=N3 bond was conjugated with the carbonyl group, the generated intermediate (IM3) was more stable than IM2, making the methyl transfer step exothermic.

### 2.5. The MD of the IM3 States

After the methyl transfer process, the elimination was supposed to occur in the typical dC-containing system to abstract the H5^dC^ and break the S-C bond. Because the methylated AZA could not undergo elimination due to the absence of hydrogen on N5, we focused on the 5′ methylated-dC (5mdC) and 5mZEB systems. In both IM3 systems, no water or ions can be found near the H5 atoms. Thus, no reagent could act as a general base to initiate the elimination.

Additionally, the IM3 of 5m-dC and 5mZEB were very stable compared to their PRS structures. Therefore, we extrapolate that a conformational change might occur to allow a general base approaching the H5, at least in the 5mdC system. Additional MD simulations were performed on the 5mdC and 5mZEB systems to study the reasonable PRS structure for the elimination.

We then interrogated the stability of the two systems by RMSD analysis again. The backbone of DNMT3A and heavy atoms in the 5mdC and 5mZEB were very stable (Figure S2) during the 1 μs MD simulations. However, the 5mZEB-containing system exhibited more stability than the 5mdC system, as the RMSD of the DNMT3A, 5mZEB, and SAH showed less fluctuation (Figure S2). Recent theoretical studies believed that a water molecule is more plausible for acting as the general base [35,44]. Thus, in order to obtain the appropriate binding pose of the water molecule for initiating the elimination, we checked the density distribution function of the oxygen atoms from the nearby water molecules to the H5 position of the 5m-dC and 5m-ZEB by the radial distribution function (RDF) in the first step (Figure S3). Two peaks of the water oxygen atoms were immediately found in both MD simulations. For instance, the first peak emerged at 3.9 Å (25.4% in the 5mdC) and 4.1 Å (14.5% in the 5mZEB), and the second appeared at 5.8 Å (27.2%) and 6.2 Å (13.2%) in the two systems. Interestingly, we found a booming tendency of oxygen atom distribution from none to 8.9% between 2.4 and 3.0 Å of the radial distance in the 5mdC system. With such a distance and water distribution, it indicated that snapshots could stand for the starting conformation to initiate the elimination. However, in the 5mZEB system, the oxygen distribution (from none to 14.5%) was observed in a distal radial range (from 3.0 to 4.1 Å). Such a distance is too far away for the water to abstract the H15^5mZEB^ from 5mZEB. Thus, it is harder for the 5mZEB to initiate the elimination.

**Figure 6:**
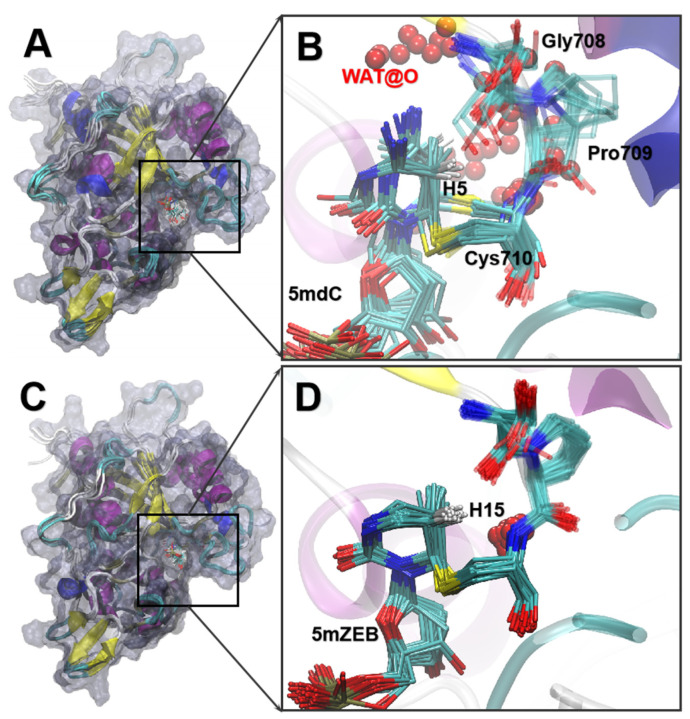
The overall structure and superimposed conformations in 5mdC (**A**,**B**) and 5mZEB (**C**,**D**) simulations. The superimposed conformations were extracted per 50 ns of the simulations. The overall systems were shown by protein secondary structure and transparent surfaces (**A**,**B**). The oxygen atoms of the nearest water molecules to H5^5mdC^/H15^5mZEB^ were shown by VDW representation. The key residues were exhibited by stick representation. The rest of the protein and DNA were displayed by cartoon representation.

The water for abstracting the H5^5mdC^/H15^5mZEB^ of the substrate also requires a specific orientation, in which the oxygen atom of the nearby water should face the C5-H5/H15 bond directly. We then extracted and superimposed the conformations (one conformation per 50 ns) and showed the oxygen atoms of the nearest water to H5^5mdC^/H15^5mZEB^ in Figure 6 to directly search the SC. In the case of the 5mdC, the base ring showed two conformational clusters that could be distinguished by *Φ*4 (C5-C6-SG-CB) (Figure S4A). Accordingly, the residues (Gly708 to Cys710) above the ring displaced a lot, while the oxygen positions of the nearest water also varied (Figure 6A). To study the two conformational clusters in the 5mdC, we extracted one conformation as the representative structure from each cluster (Figure S4B,C). In the first conformational cluster (cluster1, *Φ*4 ranges −25 to −30°), the water molecules were located aside the C5-H5 bond, indicating the cluster is inappropriate for abstracting the proton in the elimination. In the second cluster (cluster2, *Φ*4 ranges 50 to 70°), the H5 pointed toward the solvent, and the hydrogen bond between Pro709 and the amine group of the 5mdC created more room for the waters approaching H5. Thus, many oxygen atoms of the nearest water were found directly facing the C5-H5 bond. We found an oxygen atom of the nearest water molecule with 2.2 Å to H5 (Figure S4C), which is very similar to the conformations for the elimination in the former DNMT1 theoretical studies [35,44]. In contrast, the base ring of the 5mZEB was very stable, and the nearby residues were also nearly immobile (Figure 6B). The *Φ*4 was found between −24 to −29°. As a result, the oxygen distribution was observed to be densely located by the C5-H15 bond beneath the backbone of Pro709. It is, therefore, an inappropriate starting conformation for the elimination. Such positions of the nearest waters caused the two hydrogen atoms on CB^Cys^ to create a steric effect on the radial orientation of C5-H15. Moreover, the missing amine group at C4 results in H15^ZEB^ was surrounded by a series of compact backbone structures (the backbone of Gly708 and Pro709). In addition, it leaves no room for a water molecule to enter the radial space of H15^ZEB^. Therefore, the 5mZEB might fail to process the elimination because of a lack of proper water molecules.

## 3. Discussion

In this work, we performed MD simulations on the DNMT3A bound with three types of substrates (dC, AZA, and ZEB) to investigate the forming of PRS for Cys deprotonation. The results showed that the conserved Glu756 and two conserved Arginine residues (Arg792 and Arg794) stabilized the PRS structure by forming the hydrogen bonds with N3 and the O atoms in the rings of the substrates, as was previously found in DNMT1 and M. H*ha*I DNMT [35]. The PRS structure per system was similar for Cys deprotonation. We found a water molecule located at the geometrical center of the side chain of Cys710, Ser714, and the OP1 atom of the phosphate group in the substrate. Such conformation is very close to the former DNMT1 studies [35,36,43,44]. Zangi et al. [43] deemed that the H_3_O was difficult to generate because the product possessed high free energy. However, we included the phosphate group in the QM zone for the QM/MM calculations, and all three systems reported generating a hydronium ion. Aranda suggested that the Cys deprotonation directly resulted in the OP1 protonation of the adjacent base [35]. This difference might be due to the local conformational changes in the catalytic domain of DNMT1 and DNMT3A. In our DNMT3A case, the H_3_O^+^ shuttled the proton to the phosphate group in the S-C attack process, which was similar to the previous theoretical study on DNMT3A. The deprotonation of Glu756 also accompanied such a process. Therefore, we provided a new explanation for the importance of Glu756 in DNMT3A, making the ring of the substrates more positively charged to attract the newly formed thiol group of Cys710. The low Ea calculated in this step can also explain that the AZA and ZEB would swiftly form the S-C bond [45]. Additionally, we found that Glu756 protonated when the methyl group was transferred to the C5 of substrates. Thus, it also participated in the methyl transfer step. Therefore, it is more rational than Glu756 to take part in the enzymatic reactions rather than maintaining the hydrogen network for holding the ring of the substrates, as Glu756Ala nearly lost the function of methyl transfer in the previous experimental study [46].

To enlighten the synthesis of DNMT inhibitors with higher specificities, we focused on summarizing the unique inhibitory mechanisms of the AZA and ZEB presented in DNMT3A. For the AZA, two aspects were interrogated to explain the difficulties in the methyl transfer. One is that the orientation of the nucleophilic attack is unfavorable for the methyl group transfer. Another is that the more negatively charged N5 introduces stronger electrostatic repulsion to the methyl group. Thus, the AZA stops at the methyl transfer step and irreversibly seizes the DNMT3A. Such an inhibitory mechanism is similar to the ZEB in DNMT1 [36]. However, the methyl group could transfer to the ZEB with a similar Ea (21.28 kcal/mol) to the dC system. As previously postulated, the elimination in DNMT3A might be after the catalytic dynamics associated with *Φ*4 [38]. In the 5m-ZEB-containing MD, the *Φ*4 in 5m-ZEB-Cys710 was always kept between −24 to −29°. Such a conformation created a steric effect on the radial orientation of the C5-H15 as we previously notified for the range of *Φ*4 [38]. Thus, it prevents any water molecule placed near H15^ZEB^ from elimination. In this case, we argue that a methylated ZEB failed to initiate the elimination. Thus, in turn, it trapped the 5mZEB-DNA in DNMT3A as a feature of the ZEB inhibitory mechanism. Notably, our study had shown that, unlike the AZA, the ability for converting the SAM to SAH using the ZEB is similar to the nature dC, thus the ZEB might induce the decrease of the SAM. Indeed, it has been reported that men were more significantly sensitive than women to the supplementation of the SAM [47,48], and therefore, it might partly provide some evidence to understand the gender-related results of the preclinical trials of the ZEB [20]. However, more experimental evidence is needed to support this hypothesis.

## 4. Materials and Methods

### 4.1. MD Input Preparation

The initial structure of the monomer simulation (DNMT3A-DNA) was taken from the PDB:5YX2 [41]. The Cys710 and Glu756 were modeled in the protonated state, as treated in the former theoretical study of DNMT1 [35]. Similar to our previous study [49,50,51,52,53,54], the PDB2PQR server was used to determine the protonation states of amino acids under pH 7.0 for all MD systems [55]. The inputs for generating the parameters for the SAM, AZA, ZEB, 5m-dC-Cys, 5m-AZA-Cys, and 5m-ZEB-Cys, were all created by the Antechamber program of AmberTools 21 [56,57]. The RESP atomic charges [58] of these ligands were calculated at HF/6-31G* level through Gaussian 09 package [59,60]. The bond constants were obtained from the AMBER GAFF force field [61]. The proteins were defined by the AMBER 14SB force field and DNA atoms were defined by the BSC1 force field, respectively [62,63]. The counterions (Na^+^) were added to each MD system to neutralize the overall charges. The periodic solvent box with 24 Å and 16 Å TIP3P water layers [64] were added into the heterotetramer and monomer systems, respectively (Supporting Table S1).

### 4.2. MD Process

The MD processes were elaborately performed by AMBER v20 (PMEMD) with CUDA accelerate codes [56]. All the solvated systems were first minimized by 10,000 optimization steps. The MD process of each system contained the equilibrium and production phases and was repeated three times. Different initial velocities were randomly assigned to all the atoms from a Maxwell–Boltzmann distribution, as was previously undertaken on the DNMT3A systems [38]. As described in our previous studies of other receptor–ligand systems [49,50,51,52,53,54], the equilibration consisted of three stages: (a) the potential steric clashes in the initial conformation were relieved with 50,000 steps in the energy minimization; (b) each system was then heated to 300 K over 0.5 ns, with 5 kcal/mol protein harmonic constraints under the canonical ensemble (NVT) conditions; and (c) the systems were simulated for another 500 ps under the isothermal–isobaric ensemble (NPT) conditions with applied constraints gradually reduced from 10 to 0 kcal/mol. The production stage was then run at a constant temperature (300 K) and pressure (1 atm) by NPT simulations. The integration time step of the simulations was set to 2 fs, and the nonbonded cut-off length was set to 10 Å. The thermostat and barostat controls were used by Berendsen pressure compressibility at 4.57 × 10^−5^ bar^−1^ and Berendsen pressure relaxation time at 100 fs. The periodic boundary conditions (PBC) coupled with the Particle Mesh Ewald (PME) method were applied for determining the electrostatic interactions [65]. In all of the systems, in order to better mimic the system from the DNMT3A-3L physiological environment, the residues in dimer interactions were applied with the restraint factor of 10 kcal/mol/A^2^. We performed 0.2 µs × 3 replicates for the production phase to search the PRS of Cys deprotonation. For the IM3 states, we performed 1 µs simulation each on DNMT3A-5m-dC and DNMT3A-5m-ZEB, respectively. As was previously undertaken on similar MD systems [33], a clustering method was then performed to retrieve the possible PRS of Cys deprotonation upon the MD production trajectories. We monitored the same closest water molecule around both of the OP1 of dC/AZA/ZEB (OP1^dC/AZA/ZEB^). More precisely, once the H^WAT^ to SH^Cys^ and the other H^WAT^ of the same water to OP1^dC/AZA/ZEB^ were both within 1.2 to 1.8 Å, the snapshot was recognized and clustered as one of the PRS conformations. The percentage of the PRS conformation among the total production snapshots was calculated in each MD system.

### 4.3. QM/MM Simulations

The QM/MM calculations were performed with the ChemShell 3.7.0 package [66] that triggers the Gaussian 09 D.01 [60] for QM calculations and the DL_POLY program [67] for MM calculations. The QM/MM Hamiltonian was calculated by the following equations [66]:

$$E_{tot}=E\left(M,MM\right)+E\left(QL,QM\right)+E(QM({MM}^{ele})$$

The total Hamiltonian consists of molecular mechanism contribution *(*$E\left(M,MM\right)$*)*, the QM contribution ($E\left(QL,QM\right)$), and the QM/MM coupling energies ($E(QM({MM}^{ele})$). The atoms in the QM region (Q) and the link atoms (L)were described by M06-2X with 6-31(d,p) basis set, as it is better in describing the dispersion effect than B3LYP/6-31(d,p) [30,31,33]. QM/MM electrostatic interactions are calculated according to the QM electrostatic potential and MM partial charges. In applying the electrostatic embedding type of the QM/MM coupling method, the MM charges (${MM}^{ele}$) were included in the Hamiltonian of the QM part by the default setting. The cut-off of 20 Bohr was introduced for the nonbonded MM and the QM/MM interactions. The hydrogen link atoms and the charge shift model [68] were constructed to treat the QM/MM boundary in each QM/MM system. The geometry optimizations were performed using the DL_FIND program with HDLC optimizer [69] that was implemented in ChemShell. The DL-FIND optimizer [59] was performed on the selected conformations in the reactions to search for the local minima of the energy. The potential energy surfaces were then scanned along the reaction coordinates. The transition state (TS) optimization was performed at the highest energy point of the energy surface of the scan. Numerical frequency calculations on all TS structures were performed to obtain the intrinsic vibration with only one imaginary frequency for validation. The intrinsic reaction coordinate (IRC) method for QM/MM calculations was conducted here. The TS structures were verified by calculating the frequency calculations to make sure only one imaginary frequency existed. The intermediate states that connect to one TS structure were retrieved from the first negative intrinsic vibration with a scale factor of 0.2, and then optimized by DL-FIND again. The entropic effects were discarded due to the fact that they are usually minor, especially in the concurrent situation where the reactant residues (dC, AZA, and ZEB) are rigid [70,71,72].

## 5. Conclusions

**Figure 7:**
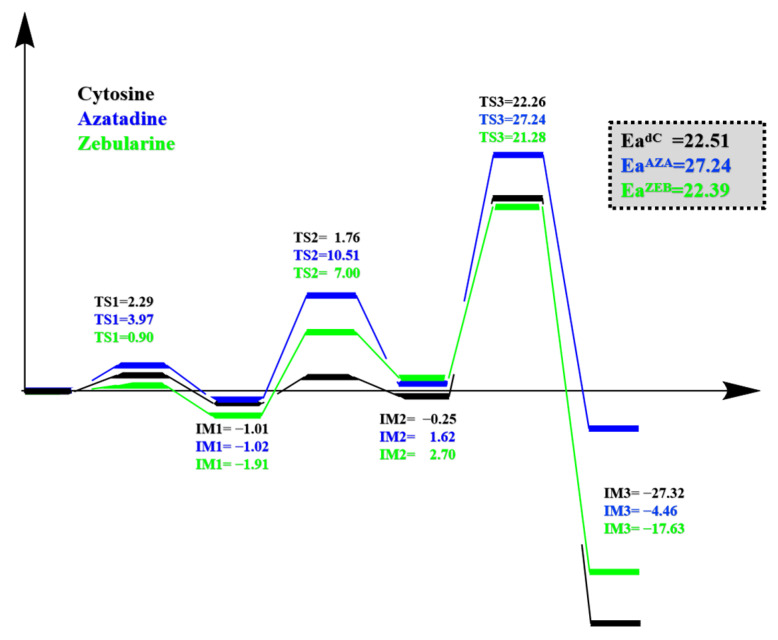
The potential energy surface of the reaction pathways linking from Cys deprotonation, S-C attack, to methyl transfer with the substrate systems Cytosine, Azatadine, and Zebularine.

In this study, we have utilized MD and QM/MM approaches to investigate the inhibitory mechanisms of the AZA and ZEB and compared them to the dC in the DNMT3A system. The potential energy surface (PES) of the major steps in Cys deprotonation, S-C attack, and methyl transfer is shown in Figure 7. Our computational results indicate that the AZA could process the Cys deprotonation and S-C attack pathways while stopping the methyl transfer reaction. In comparison, the ZEB might process the methyl transfer, but would be stuck in a failure conformation for the unique inhibitory mechanism. We think future improvements of ZEB might need to focus on the C5 modifications to disable it to react with the SAM. In this way, the new inhibitor might bring a similar drug performance to different genders. We anticipate that the findings might contribute to a better understanding of the inhibitor mechanism of DNMT3A, thus shedding light on the future drug design of DNMTi.

## Data Availability

The data presented in this study are available in Supplementary Material (CF.pse file).

## Supplementary Materials

The following supporting information can be downloaded at: https://www.mdpi.com/article/10.3390/ijms241612652/s1. Reference [73] is cited in the supplementary materials.
