# Supplementary material for: Insights into the Inhibitory Mechanisms of the Covalent Drugs for DNMT3A
Source: Int J Mol Sci. 2023 Aug 10;24(16):12652. doi: 10.3390/ijms241612652 (PMC10454219; doi:10.3390/ijms241612652)
Supplement: Supplementary file 1 [file ijms-24-12652-s001.zip › supporting information-v5-final.pdf]

Supporting Information

Insights into the inhibition mechanism of the covalent drugs for DNMT3A

Wei Yang [1,2], Jingyuan Zhuang [1], Chen Li [3], Chen Bai [1,4,5,*] and Gui-Juan Cheng [1,4,5,6,*]

[1] Warshel Institute for Computational Biology, School of Medicine, The Chinese University of Hong Kong, Shenzhen, Shenzhen 518172, China; yangwade86@gmail.com (W.Y.); zhuangjingyuan08@gmail.com (J.Z.)
[2] National Clinical Research Center for Infectious Diseases, Shenzhen Third People's Hospital, Shenzhen 518112, China
[3] Biomedicine Discovery Institute, Department of Biochemistry and Molecular Biology, Monash University, Melbourne, VIC 3800, Australia; chen.li@monash.edu
[4] School of Life and Health Sciences, The Chinese University of Hong Kong, Shenzhen, Shenzhen 518172, China
[5] School of Medicine, The Chinese University of Hong Kong, Shenzhen, Shenzhen 518172, China
[6] Shenzhen Futian Biomedical Innovation R&D Center, The Chinese University of Hong Kong, Shenzhen,Shenzhen 518017, China

QM calculations of model systems: Three trimmed systems were modeled to study the preferred attack angle of the methyl group in the nucleophilic addition step. The initial TS structures of dC and AZA-2 systems were established based on the QM/MM optimized TS3 structures of AZA and dC-containing systems. The initial TS structure of the AZA-1 system was built manually. The transition states and IM2 were optimized in water using the B3LYP/6-31G* method. All the QM calculations were performed using the Gaussian 16[1] software.

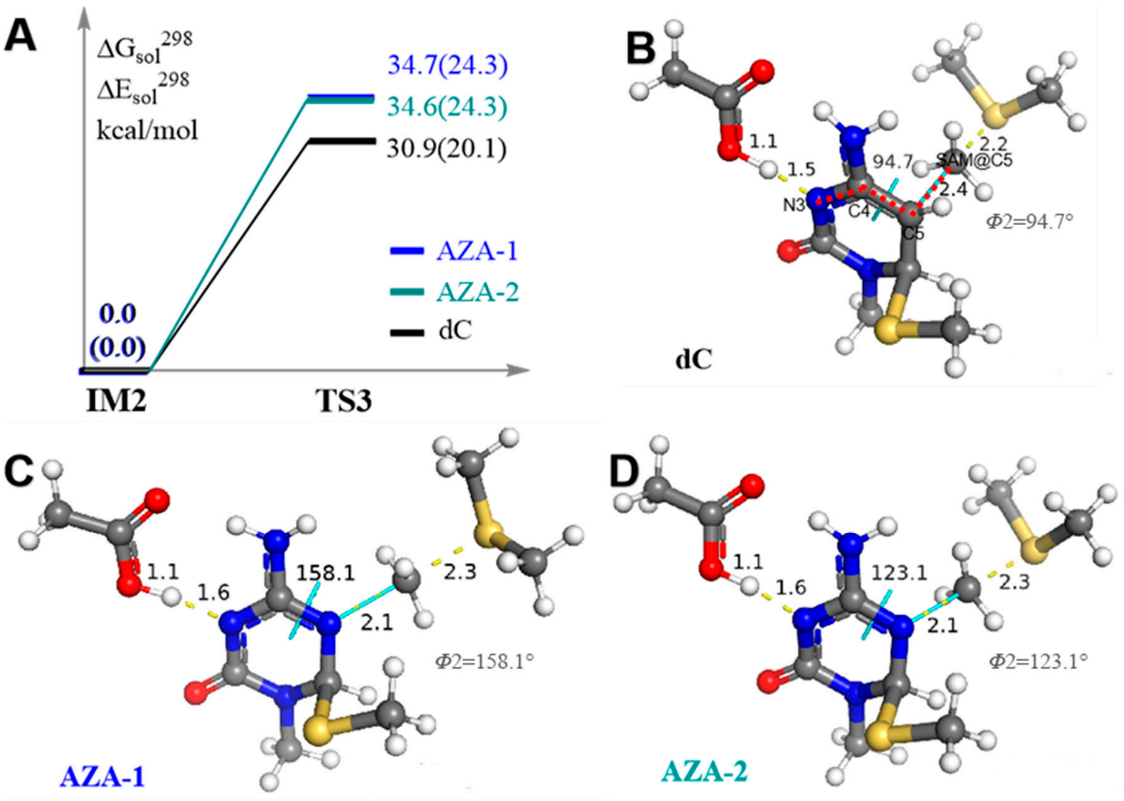

Figure S1. (A) The energy profiles and (B-D) optimized TS structures for the model systems. The $\Phi 2$ (N3-C4-C5-SAM@C5) with cyan dihedral representations were indicated separately under Panels B-D. The key distances were annotated using yellow dashes.

Table S1. The detailed key distances and dihedral from reactant state to IM3 in the DNMT3A-DNA^{dC} system (unit=Å, dihedral unit=°).

	Distance detail	R	TS1	IM1	TS2	IM2	TS3	IM3
d1	Cys710@SG-Cys710@HG	1.37	1.61	1.93	2.24	2.43	2.51	2.59
d2	Cys710@HG-WAT@O	1.77	1.26	1.05	0.99	0.97	0.97	0.97
d3	WAT@H2-dC304@OP1	1.84	1.67	1.44	1.24	1.06	1.06	1.05
d4	Cys710@SG-dC304@C6	3.48	3.44	3.39	2.34	2.02	1.99	1.88
d5	Glu756@ HE2-Glu756@OE2	1.04	1.04	1.04	1.36	1.64	1.18	1.03
d6	dC304@ N3 -Glu756@HE2	1.62	1.61	1.6	1.29	1.06	1.4	1.68
d7	dC304@C5-SAM@C5	3.27	3.31	3.33	3.37	3.38	2.31	1.54
d8	SAM@C5-SAM@S	1.80	1.81	1.81	1.8	1.81	2.28	3.38
*Φ*1	dC304@H6-C6-C5-N1	0.92	0.92	1.35	-31.27	-43.06	-61.17	-80.72
*Φ*3	SAM@H7-C5-H8-H9	-125.9	-126	-125.67	-124.41	-124.1	-172.62	118.76

Table S2. The detailed key distances and dihedral from reactant state to IM3 in the DNMT3A-DNA^{AZA} system (unit=Å, dihedral unit=°).

	Distance detail	R	TS1	IM1	TS2	IM2	TS3	IM3
d1	Cys710@SG-Cys710@HG	1.37	1.55	1.96	2.11	2.43	2.47	2.58
d2	Cys710@HG-WAT@O	1.51	1.31	1.04	1.00	0.97	0.97	0.97
d3	WAT@H2-**AZA@OP1**	1.59	1..56	1.35	1.15	1.06	1.06	1.05
d4	Cys710@SG-**AZA@C6**	3.34	3.33	3.17	2.46	1.95	1.96	1.90
d5	Glu756@HE2-Glu756@OE2	1.02	1.02	1.04	1.41	1.61	1.07	1.03
d6	**AZA@N3**-Glu756@HE2	1.71	1.70	1.62	1.14	1.07	1.54	1.65
d7	**AZA @N4**-SAM@C5	3.27	3.27	3.17	3.27	3.03	2.16	1.49
d8	SAM@C5-SAM@S	1.80	1.80	1.80	1.80	1.81	2.27	3.22
Φ1	**AZA @H6-C6-N4-C5**	178.56	-179.43	-178.71	162.37	136.08	159.01	167.90
Φ3	SAM@H7-C5-H8-H9	-124.51	124.53	-124.61	-124.75	-125.15	-173.15	119.02

Table S3. The detailed key distances and dihedral from reactant state to IM3 in the DNMT3A-DNA^{ZEB} system (unit=Å, dihedral unit=°).

	Distance detail	R	TS1	IM1	TS2	IM2	TS3	IM3
d1	Cys710@SG-Cys710@HG	1.35	1.54	1.97	2.25	2.47	2.47	2.58
d2	Cys710@HG-WAT@O	1.89	1.32	1.03	0.98	0.97	0.97	0.97
d3	WAT@H2- **ZEB@OP1**	2.00	1.56	1.36	1.09	1.06	1.05	1.05
d4	Cys710@SG-**ZEB@C6**	3.50	3.38	3.20	2.37	1.94	1.95	1.88
d5	Glu756@HE2-Glu756@OE2	1.03	1.02	1.03	1.42	1.53	1.06	1.01
d6	**ZEB@N3**-Glu756@HE2	1.6	1.68	1.6	1.11	1.08	1.54	1.71
d7	**ZEB@C5-**SAM@C5	3.34	3.40	3.32	3.32	3.34	2.29	1.56
d8	SAM@C5-SAM@S	1.81	1.80	1.80	1.80	1.81	2.30	3.42
Φ1	**ZEB@H6-C6-C5-N1**	-178.24	-177.90	-178.18	147.63	125.68	140.35	153.68
Φ3	SAM@H7-C5-H8-H9	-125.37	-124.76	-125.15	-124.20	-124.78	-174.46	118.00

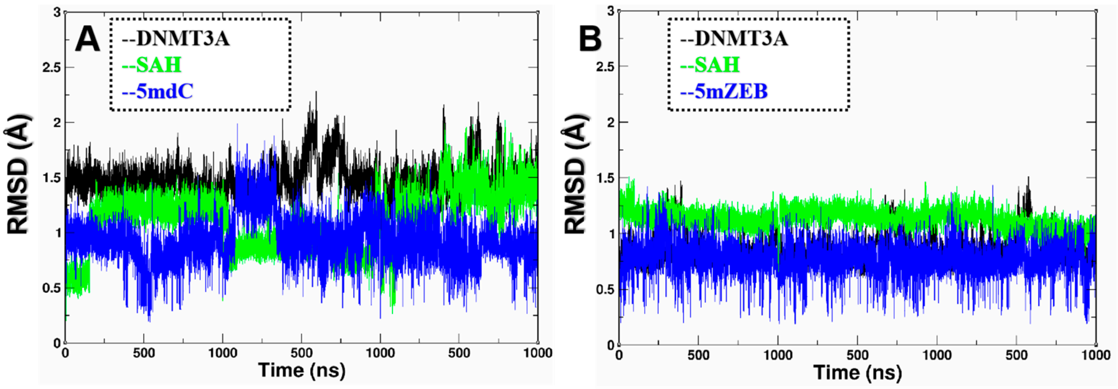

Figure S2. The RMSD of the IM3 structures in (A) 5mdC and (B) 5mZEB containing systems.

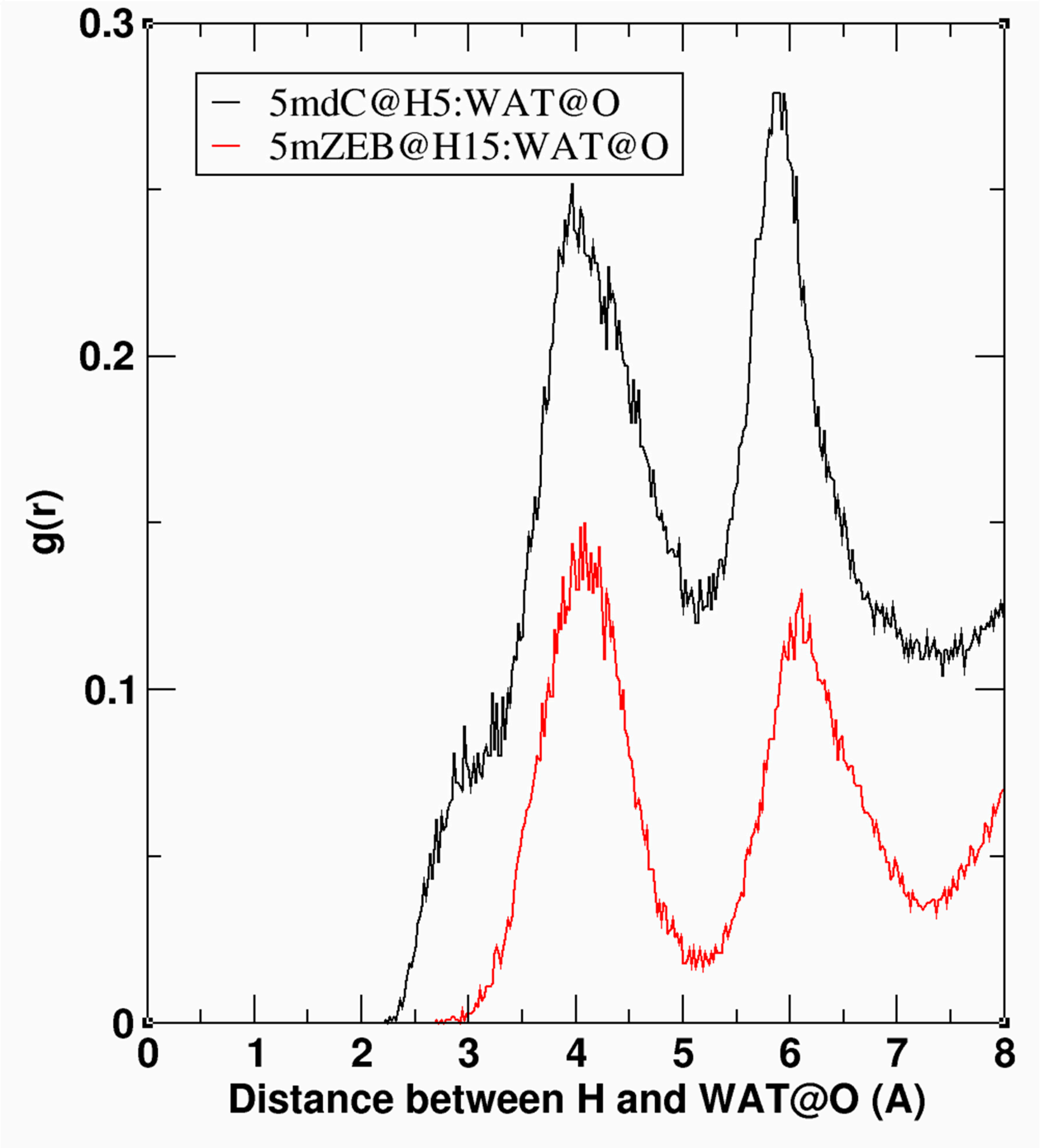

Figure S3. The RDF plot for the two systems. The X-axis refers to the distance between the oxygen atom of the water molecule and the H5 atom of (A) 5m-dC and (B) 5m-ZEB, respectively. The Y-axis presents the radial distribution function of the oxygen atoms.

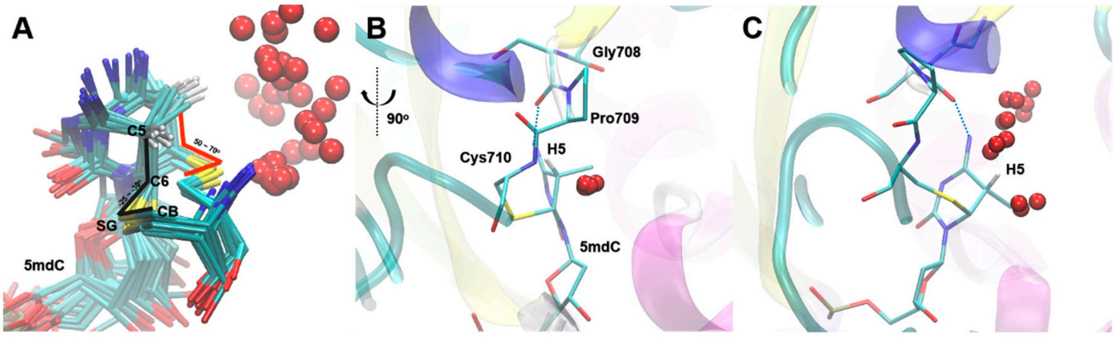

Figure S4. The two conformational clusters of 5mdC-Cys710 found in the 5mdC simulation. (A) The detailed structure information shown by the superimposed structures. Two clusters were distinguished by the dihedral $\Phi 4$ (C5-C6-SG-CB) and shown by black (cluster1) and red (cluster2) lines, respectively. The oxygen atoms of the nearest water molecules to H5 of 5mdC were shown by the VDW representation. The representative structures from cluster1 and cluster2 were shown in panels B and C (rotated 90° from panel B). The cluster oxygen atoms of the nearest water molecules were shown per panel B and C for clarity.

Supporting Coordinate File (CF): The coordinate file of all reactants, products, IMs, and TSs calculated in this work were stored in the CF.pse file, which can be opened by Pymol (https://pymol.org/2/).

Reference:

1. Gaussian 16, Frisch, M. J.; Trucks, G. W.; Schlegel, H. B.; Scuseria, G. E.; Robb, M. A.; Cheeseman, J. R.; Scalmani, G.; Barone, V.; Petersson, G. A.; Nakatsuji, H.; Li, X.; Caricato, M.; Marenich, A. V.; Bloino, J.; Janesko, B. G.; Gomperts, R.; Mennucci, B.; Hratchian, H. P.; Ortiz, J. V.; Izmaylov, A. F.; Sonnenberg, J. L.; Williams-Young, D.; Ding, F.; Lipparini, F.; Egidi, F.; Goings, J.; Peng, B.; Petrone, A.; Henderson, T.; Ranasinghe, D.; Zakrzewski, V. G.; Gao, J.; Rega, N.; Zheng, G.; Liang, W.; Hada, M.; Ehara, M.; Toyota, K.; Fukuda, R.; Hasegawa, J.; Ishida, M.; Nakajima, T.; Honda, Y.; Kitao, O.; Nakai, H.; Vreven, T.; Throssell, K.; Montgomery, J. A. Jr., Peralta, J. E.; Ogliaro, F.; Bearpark, M. J.; Heyd, J. J.; Brothers, E. N.; Kudin, K. N.; Staroverov, V. N.; Keith, T. A.; Kobayashi, R.; Normand, J.; Raghavachari, K.; Rendell, A. P.; Burant, J. C.; Iyengar, S. S.; Tomasi, J.; Cossi, M.; Millam, J. M.; Klene, M.; Adamo, C.; Cammi, R.; Ochterski, J. W.; Martin, R. L.; Morokuma, K.; Farkas, O.; Foresman, J. B.; and Fox, D. J. Gaussian, Inc., Wallingford CT, 2016.
